# Cycling on a Bike Desk Positively Influences Cognitive Performance

**DOI:** 10.1371/journal.pone.0165510

**Published:** 2016-11-02

**Authors:** Tine Torbeyns, Bas de Geus, Stephen Bailey, Kevin De Pauw, Lieselot Decroix, Jeroen Van Cutsem, Romain Meeusen

**Affiliations:** 1 Human Physiology Research Group, Vrije Universiteit Brussel, Brussels, Belgium; 2 Department of Physical Therapy Education, Elon University, Elon, United States of America; 3 School of Public Health, Tropical Medicine and Rehabilitation Sciences, James Cook University, Queensland, Australia; University of Akron, UNITED STATES

## Abstract

**Purpose:**

Cycling desks as a means to reduce sedentary time in the office has gained interest as excessive sitting has been associated with several health risks. However, the question rises if people will still be as efficient in performing their desk-based office work when combining this with stationary cycling. Therefore, the effect of cycling at 30% Wmax on typing, cognitive performance and brain activity was investigated.

**Methods:**

After two familiarisation sessions, 23 participants performed a test battery [typing test, Rey auditory verbal learning test (RAVLT), Stroop test and Rosvold continuous performance test (RCPT)] with electroencephalography recording while cycling and sitting on a conventional chair.

**Results:**

Typing performance, performance on the RAVLT and accuracy on the Stroop test and the RCPT did not differ between conditions. Reaction times on the Stroop test and the RCPT were shorter while cycling relative to sitting (p < 0.05). N200, P300, N450 and conflict SP latency and amplitude on the Stroop test and N200 and P300 on the RCPT did not differ between conditions.

**Conclusions:**

This study showed that typing performance and short-term memory are not deteriorated when people cycle at 30% Wmax. Furthermore, cycling had a positive effect on response speed across tasks requiring variable amounts of attention and inhibition.

## Introduction

It has been shown that physical activity [1] is associated with several aspects of a long and high-quality life. People with higher physical activity levels show lower risks of developing metabolic syndrome, cardiovascular disease, diabetes, some types of cancer, hypertension, obesity and mental health problems [2]. Higher levels of physical activity seem to also be associated with a better quality of life, lower stress levels, better social interaction and a better self-perception [3, 4]. Furthermore, physical activity is known to enhance executive functions [3, 5]. However, recent studies suggest that sedentary behaviour [6] is even more important in determining health, showing that overall sedentary time is associated with risk factors for cardiometabolic disease, some cancers and mortality [7].

Prolonged sitting has been engineered into our lives across many settings, including transportation, the workplace, schools and the home [7]. Most people spend at least eight hours per day at work, and more and more have sedentary occupations [8]. Therefore, focus on reducing work-associated sitting behaviour is needed, e.g. the implementation of active workstations at the workplace. Providing walking desks in the office leads to a reduced time spent in sedentary behaviours, an increase in daily step count and energy expenditure, and has a positive effects on several health parameters [9]. However, the question rises if people will still be as efficient in performing their desk-based office work when combining this with stationary walking or cycling. In the study of Straker et al. [10], a slight decrease in computer task performance was observed, while using a treadmill and a bike desk. Also in the study of Koren et al. [11] a slight negative effect of cycling on typing performance was observed. In comparison, Elmer et al. [12] and Commissaris et al. [13] found no effect on typing performance while cycling. Furthermore, in most studies, no effect on selective attention and processing speed, response inhibition, divided attention, short-term auditory verbal memory, perceptual performance, executive memory, working memory, math, vocabulary and reasoning while cycling and walking was reported [11, 13–17]. In the study of Pontifex et al. [18], a detrimental effect of cycling on selective attention was seen. However, familiarisation periods were limited in these aforementioned studies, with the longest familiarisation period being 15 minutes [16]. It could be that participants were not yet completely familiar to the new dual task of combining exercise and computer tasks. Therefore, it would be of interest to investigate if the effect of cycling on typing and cognitive task performance would be different when people have the possibility to adapt to the dual task for a longer period of time.

Cognitive tests selected in previous research were often directed to measuring short term memory [15, 19], selective attention and response inhibition [13–17]. Tests to measure these parameters that have been used in previous active workstation research are the Rey auditory verbal learning test to measure short term memory [19] and the Stroop test to measure selective attention and response inhibition [14–16]. Furthermore, we were interested if cycling could contribute to maintaining a higher sustained attention, as with the current eight hour work day, it is important to stay focused for a long time. Therefore, we selected the Rey auditory verbal learning test to measure short term memory, the Stroop test to measure selective attention and response inhibition, and the Rosvold continuous performance test to measure sustained attention.

It is of interest to also look into neuronal processes underlying cognitive performances as these might be altered when cognitive challenge is combined with physical activity. Event-related potentials (ERPs) are one of the most informative and dynamic methods of monitoring brain activity and provide a high temporal resolution [20]. The ERP is characterized by a succession of positive and negative components [21]. Two components of the ERP which bear special importance to stimulus evaluation, selective attention, response inhibition and conscious discrimination are the N200 and P300 [22]. N200 amplitude is associated with response inhibition during tasks that elicit conflict, with increased amplitude reflecting greater conflict monitoring [23]. N200 latency is suggested to be positively related to reaction time [24]. Furthermore, P300 latency corresponds to the speed of cognitive processing and the amplitude shows the allocation of brain energy resources [21]. A shorter P300 latency reflects a shorter stimulus evaluation time, while a larger P300 amplitude indicates more attentional resources devoted to a given task [25]. Two other ERP components playing a role in conflict detection, resolution and response selection and especially of interest during the Stroop task are the N450 and the conflict slow potential (conflict SP). The N450 is shown to be more negative following incongruent trials than following congruent trials. For the conflict SP, more positive amplitudes have been associated with increased response times and accuracy [26]. To our knowledge only one study looked into ERPs during low intensity cycling. The findings of that study [18] suggested that the need to allocate attentional resources toward the bodily movements associated with exercise may relate to inefficiency of neural resource allocation, resulting in decreased interference control. However, also in this case, it would be of interest to investigate if the effect of cycling on these neuronal processes would be different when people have the possibility to adapt to the dual task for a longer period of time.

The aim of this study was to assess the participants’ typing and cognitive performance while cycling and sitting. Electrophysiological measurements to evaluate neuronal processes were performed during the cognitive tests. Because of the familiarisation sessions, it was hypothesised that typing would not be influenced while cycling. Furthermore, we expected an improved cognitive performance and facilitated brain function during the cognitive tests in the cycling condition because of an increased state of vigilance and an exercise-related increase in arousal.

## Materials and Methods

### Participants

A priori power calculation, performed with G*Power 3.1.9.2, revealed that a minimum of 23 participants was needed to achieve sufficient power. This sample size calculation was based on the effect sizes for two main outcome measures of this study, namely typing and response inhibition, reported by Larson et al. [17, 19].

Therefore, we included twenty-three volunteers (7 male, 16 female) in this study. Participants were recruited via online advertisements and leaflets at the Vrije Universiteit Brussel, Belgium. To be included in this study subjects were required to have a sedentary occupation (min 70% of the workday) and to participate in moderate to vigorous physical activity for a maximum of 2.5 hours per week. Exclusion criteria were the presence of attention deficit hyperactivity disorder, type 1 and type 2 diabetes, cardiovascular disease, depression, musculoskeletal problems, use of stimulants and beta blockers. The study was approved by the Ethical Committee of the UZ Brussel and the Vrije Universiteit Brussel (B.U.N. 143201318930). Written informed consent was obtained from all individual participants included in the study.

### Procedures

#### Incremental cycle test to exhaustion and familiarisation sessions

Participants visited the laboratory five times. During their first visit, they filled out a questionnaire concerning physical activity status (International Physical Activity Questionnaire [27]), underwent a medical screening by a medical doctor, and performed an incremental cycle test to exhaustion. To minimise the learning effect, this test session was followed by two familiarisation sessions, taking place on two consecutive days. During these familiarisation sessions, the participants practised the cognitive test battery while sitting on a conventional chair (familiarisation session 1) and while cycling on the bike desk (familiarisation session 2). Furthermore, during both familiarisation sessions, participants were asked to cycle for 30 minutes, at 30% Wmax, in combination with their normal work activity. This way, the participants had the opportunity to become familiar with using the bike desk.

#### Test sessions

One week after familiarisation session 2, the participants returned to the lab twice, once to perform the cognitive test battery while sitting on a conventional chair, and once while cycling at 30% of their maximal external power (Wmax) on the bike desk. These two test moments were counterbalanced and were conducted one week apart at the same time of the day (between 9 and 12 am) to avoid influence of the circadian rhythm. Participants were not allowed to consume alcohol and to engage in vigorous physical activity 24 hours before the test moment. Furthermore, they were asked to consume a prescribed breakfast. Caffeine consumption on the test days was not allowed.

At the start of the test sessions, participants were fitted with a polar heart rate monitor and transmitter (Polar X-Trainer Plus, Polar Electro OY, Kempele, Finland) and the electroencephalography (EEG) cap (Acticap, Brain Products, Munich, Germany) was attached. This was followed by a practice Stroop test and typing performance trial. During these practice trials, computerised feedback about accuracy and speed was given. Subsequently, the participants performed a test battery consisting of the ‘Rey auditory verbal learning test’ (RAVLT), the ‘Stroop test’ with electroencephalography (EEG) recording, a typing test and the ‘Rosvold continuous performance test’ (RCPT) with EEG recording. These tests are described in detail below. The test battery took about 30 minutes. Participants continuously cycled, without any breaks.

#### Bike desk

The desk of the in height adjustable LifeSpan C3-DT5 Bike Desk was combined with an electrically braked cycle ergometer (Excalibur Lode, Groningen, the Netherlands). Power output was delivered in an isoinertial way (constant power), meaning that participants simply had to pedal at a self-selected pedalling rate without having to pay attention to hitting the target power output of 30% Wmax. Participants adjusted the cycle ergometer and the table until they were sitting in a comfortable posture. They had the possibility to re-adjust the cycle ergometer and the table after the practice Stroop and typing trial.

#### Participants’ characteristics

Age was self-reported. Height, body weight and fat percentage were measured to the nearest 0.1 cm, 0.1 kg and 0.1%, respectively. Peak oxygen uptake capacity (VO_2_peak) was measured using an indirect calorimetry system (Metalyzer II^®^, Cortex Biophysik, Leipzig, Germany) during an incremental cycle test to exhaustion. Participants started cycling at 50 Watts. Every three minutes, the load increased 25 Watts. The participants were asked to maintain a constant rhythm of 80 rpm. They were encouraged to exert themselves until volitional exhaustion. The decision to stop was based on signals of extreme fatigue and was confirmed by a heart rate that approximated the theoretical maximum heart rate (220-age) or a respiratory exchange ratio >1.10. VO_2_peak was defined as the highest VO_2_ attained over 30 sec. The maximal exercise test was performed on an electrically braked cycle ergometer (Excalibur Lode, Groningen, the Netherlands). Heart rate and rate of perceived exertion were assessed during the cognitive test batteries. Heart rate was recorded every 30 seconds using a polar heart rate monitor and transmitter (Polar X-Trainer Plus, Polar Electro OY, Kempele, Finland). Participants were asked to indicate their Rate of Perceived Exertion (RPE) on a modified 10-point Borg scale (Borg, 1982) at the end of both test batteries.

#### Transcription test

The transcription test was performed using ‘TypingMaster Pro’ (TypingMaster, Inc., Helsinki, Finland). Participants were provided with a different text of similar difficulty during each test moment. The order of both texts was counterbalanced. The participants were asked to transcribe as much of the text as possible within five minutes, while making as few mistakes as possible. Participants could not rely on spell check and could not go back in the typed text. They could only correct within the word they were typing. Typing speed and the number of mistakes were registered. Performance on the test was expressed in adjusted words per minute (AWPM) [14, 28].

#### Rey auditory verbal learning test

The Dutch (native speech of the participants) version of the Rey auditory verbal learning test was used to assess short-term memory [19]. Fifteen words were five times read aloud by a trained staff member. Participants were each time asked to recall as many words as possible. After 20 minutes, at the end of the test battery, participants were asked to recall as many words as possible and to recognise the words of the list within a list of 30 read aloud words. The sum of recalled words of the five first trials, the amount of recalled words of the recall session and the amount of correctly and incorrectly recognised words during the recognition trial were used as outcome measures.

#### Stroop test

The Stroop test was programmed and performed on E-prime 2.0 software (Psychology Software Tools, Inc., Pittsburg, PA). The Stroop test was used to measure selective attention and response inhibition [29, 30]. The test consisted of three parts [16]. In the first part, measuring neutral reaction time, participants were demonstrated with X’s coloured in yellow, red, blue and green, and were asked to respond by pushing the corresponding button on a keyboard (AZERTY; F—left middle finger, V—left index finger, B—right index finger, H—right middle finger). In the second part, the words yellow, red, blue and green were shown in matching colours (congruent condition) and non-matching colours (incongruent condition). Participants were asked to push the button corresponding to the colour in which the words were displayed. In the third part, again the words yellow, red, blue and green were shown in matching colours (congruent condition) and non-matching colours (incongruent condition). This time, the participants were asked to push the button corresponding to the word displayed on the screen. The three parts were separated by a 30-sec rest period. Sixty stimuli were presented in the first part, 60 congruent and 60 incongruent in the second part, and 60 congruent and 60 incongruent in the third part. The interval response—stimulus onset was set at 500ms. The stimuli were displayed in the middle of the computer screen. The total time to perform the three parts of the test was approximately four minutes. Outcome measures were accuracy (%) and reaction time (ms).

#### Rosvold continuous performance test

The Rosvold continuous performance test (RCPT) was programmed and performed on E-prime 2.0. software (Psychology Software Tools, Inc., Pittsburg, PA). The RCPT was used to measure sustained attention [29, 31]. Over a time span of seven minutes, letters were continuously (every 1000 ms) presented to the participants. The participants were asked to push the space bar when an X appeared on the screen. Accuracy (%) and reaction time (ms) were assessed.

#### Electrophysiological measurements

Continuous EEG was registered during the Stroop test and the RCPT using BrainVision Recorder (Brain Products GmbH, Munich, Germany). EEG data were derived from 32 active Ag/AgCl electrodes attached on the subjects’ head (Acticap, Brain Products, Munich, Germany) according to the ‘10-20 International System’ [32]. Electrode impedance was kept < 5k*Ω* throughout the experiment. Continuous data were recorded at a sampling rate of 500 Hz. To minimise sound artefacts, subjects were provided with earplugs.

ERP data were analysed using BrainVision Analyzer (Brain Products GmbH, Munich, Germany). Latency (ms) and amplitude (*μ*V) were assessed for the N200, P300, N450 and conflict SP in response to the different stimuli during the Stroop test and for the N200 and P300 in response to the RCPT. The ERP data were processed as follows. Raw data were filtered (high pass: 0.1 Hz, low pass: 45 Hz and notch: 50 Hz; slope: 48dB/oct) with a Butterworth filter design and re-referenced to an average reference. Subsequently, artefacts were removed semi-automatically. Gradient, max-min, amplitude and low activity were set at 75 *μ*V/ms, 150 *μ*V/200ms, -100 *μ*V, +100 *μ*V and 0.5 *μ*V/50ms respectively. Thereafter, the dataset was segmented (-200ms pre to 800ms post stimulus) into stimulus-locked epochs for correct trials. For each stimulus-locked epoch, artefacts were further removed using Independent Component Analysis (ICA) and inverse ICA. Baseline correction (using pre stimulus period -200 ms to 0 ms) was applied, the epochs were averaged, and N200, P300, N450 and SP were automatically detected. The number of trials contributing to the averages used in further analysis can be found in Table 1. The latency and amplitude for each ERP component were quantified using the mean amplitude and corresponding latency within a 150-300ms latency window for the N200, a 250-500ms latency window for the P300, a 450-550ms latency window for the N450 and a 600-800ms latency window for the conflict SP [18, 33–35]. Thereafter, the BrainVision Analyzer data were exported to IBM SPSS Statistics 22 for further analyses. The N200 and the N450 emerge fronto-centrally, while the P300 and the conflict SP emergs in temporal-parietal areas [18, 36, 37]. Therefore, we used the fronto-central region including Fp1, Fp2, F4, Fz, F3, F7, F8, FC1 and FC2 to analyse the N200 and the N450 and the temporal-parietal region including Pz, P3, P4, P7, P8, PO9 and PO10 to analyze the P300 and the conflict SP.

**Table 1:** Number of trials contributing to the averages used in ERP analysis.

Cognitive task		Sitting condition	Cycling condition
Stroop test	Simple stimuli	50 ± 6	47 ± 9
	Colour Congruent stimuli	45 ± 13	42 ± 15
	Colour Incongruent stimuli	34 ± 17	34 ± 18
	Word Congruent stimuli	49 ± 11	44 ± 14
	Word Incongruent stimuli	42 ± 9	39 ± 14
Rosvold continuous performance test		75 ± 8	62 ± 16

### Data analyses

Statistical analyses were conducted using IBM SPSS statistics 22. The one-sample Kolmogorov-Smirnov test was used to test the normality of the data. Data are presented as mean ± SD. Statistical significance was set at p < 0.05.

Typing performance, performance on the RAVLT, the RCPT and the ERP data measured during the RCPT in the sitting and the cycling condition were compared with a repeated measures ANOVA in which condition (sitting, cycling) was used as within factor. To compare reaction times and ERP data on the Stroop test, a 2 (condition: sitting, cycling) x 5 (stimulus type: neutral, colour congruent, colour incongruent, word congruent, word incongruent) repeated measures ANOVA was used. If a significant interaction effect in the two-way repeated measures ANOVA was observed, subsequent paired t-tests were performed in order to interpret the effect of condition (sitting vs. cycling) for each stimulus type. If no significant interaction effect in the two-way repeated measure ANOVAs was observed, main effects were immediately observed and further interpreted through pairwise comparisons with Bonferroni correction. Sphericity was verified by the Mauchly’s test. When the assumption of sphericity was not met, the significance of F-ratios were adjusted with the Greenhouse-Geisser procedure. Furthermore, interference scores (incongruent—congruent) on the Stroop task were compared using a repeated measures ANOVA in which condition (sitting, cycling) was used as within factor. Partial-eta^2^ $\eta_{\text{p}}^{2}$ is reported as a measure of effect size for ANOVA analyses. The accuracy data of the Stroop test were not normally distributed. Therefore, Wilcoxon signed ranks tests were used to investigate differences between the cycling and the sitting condition. Differences in reaction time on the RCPT were investigated using a repeated measures ANOVA with condition (sitting, cycling) as within factor. The accuracy data of the RCPT were not normally distributed. Therefore, Wilcoxon signed ranks tests were used to investigate differences between the cycling and the sitting condition.

## Results

### Participants’ characteristics

All participants (age, 35.7 ± 10.3 years; height, 170.4 ± 6.2 cm; body weight, 67.1 ± 8.6 kg; BMI, 23.2 ± 3.0 kg/m^2^; VO_2_peak, 37.2 ± 7.7 ml/kg/min; HRmax, 183.9 ± 9.2 bpm) completed the study. Peak power output reached during the maximal exercise test was 183.7 ± 45.6 Watt and thus participants cycled at a power output of 55.3 ± 13.6 Watt during the experimental cycling condition. Heart rate and RPE were significantly higher in the cycling condition than in the sitting condition (116.8 ± 16.0 vs. 78.5 ± 11.7 bpm, p < 0.001; 3.3 ± 1.1 vs. 2.3 ± 1.0, p = 0.004).

### Transcription test

Typing performance (AWPM) did not significantly differ between the sitting and the cycling condition (F(1,22) = 0.556, p = 0.464, $\eta_{\text{p}}^{2}=0.025$). Mean ± SD values can be found in Table 2.

**Table 2:** Mean ± SD values for performances on the typing test, RAVLT, Stroop test, RCPT.

		Sitting condition	Cycling condition
**Typing test**	Adjusted words per minute (n)	44.3 ± 11.2	43.7 ± 12.6
**RAVLT**	Repeated words (n)	53.4 ± 10.0	54.6 ± 8.9
	Recalled words (n)	10.3 ± 3.1	9.9 ± 3.2
	Correctly recognised words (n)	13.5 ± 2.0	13.2 ± 2.3
	Incorrectly recognised words (n)	0.5 ± 0.8	0.5 ± 1.3
**Stroop test**	ACC Neutral stimuli (%)	96.1 ± 3.7	96.0 ± 3.7
	ACC Colour Congruent stimuli (%)	97.8 ± 3.6	97.8 ± 3.3
	ACC Colour Incongruent stimuli (%)	94.9 ± 6.2	94.5 ± 5.0
	ACC Word Congruent stimuli (%)	97.8 ± 3.2	98.0 ± 2.8
	ACC Word Incongruent stimuli (%)	95.4 ± 6.0	95.5 ± 3.7
	RT Neutral stimuli (ms)	586.0 ± 80.6	571.1 ± 95.2
	RT Colour Congruent stimuli (ms)	635.0 ± 134.3	607.9 ± 108.0
	RT Colour Incongruent stimuli (ms)	766.3 ± 203.6	748.6 ± 197.6
	RT Word Congruent stimuli (ms)	623.8 ± 96.8	603.4 ± 87.7
	RT Word Incongruent stimuli (ms)	704.4 ± 111.4	666.8 ± 113.8
**RCPT**	ACC (%)	100.0 ± 0.2	99.8 ± 0.4
	RT (ms)	404.3 ± 36.4	377.9 ± 27.7

ACC accuracy, RAVLT Rey auditory verbal learning test, RCPT Rosvold continuous performance test, RT reaction time

### Rey auditory verbal learning test

The amount of immediately repeated words (F(1,22) = 0.644, p = 0.431, $\eta_{\text{p}}^{2}=0.028$), the recalled words (F(1,22) = 0.511, p = 0.482, $\eta_{\text{p}}^{2}=0.023$) and the correctly and incorrectly recognised words (F(1,22) = 1.131, p = 0.299, $\eta_{\text{p}}^{2}=0.049$; F(1,22) = 0,000, p = 1.000, $\eta_{\text{p}}^{2}=0.000$), did not significantly differ between both conditions. Mean ± SD values can be found in Table 2.

### Stroop test

**Fig 1:**
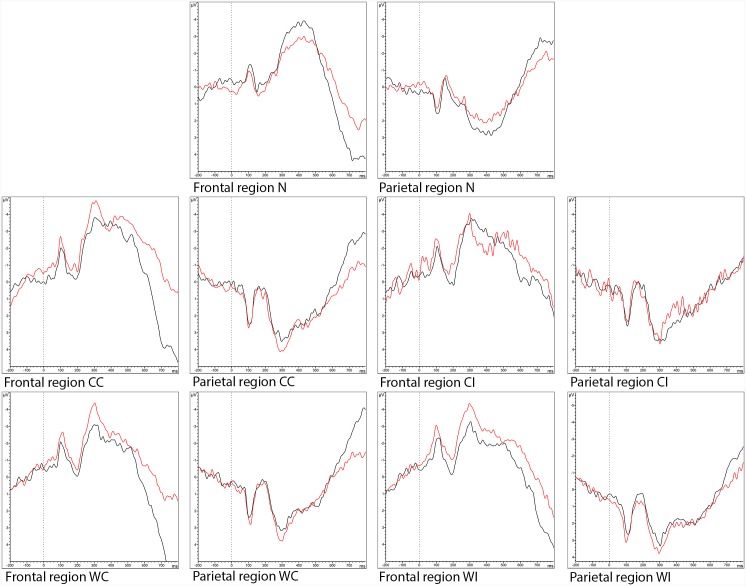
Grand averaged stimulus-locked ERP waveforms in the frontal (Fp1, Fp2, F4, F3, Fz, F7, F8, FC1, FC2) and the parietal region (P3, P4, Pz, P7, P8, PO9, PO10) region in response to the neutral (N), colour congruent (CC), colour incongruent (CI), word congruent (WC) and word incongruent (WI) stimuli of the Stroop test in the sitting (black line) and the cycling (red line) condition.

Accuracy did not significantly differ between the sitting and the cycling condition. A significant main effect of condition (F(4,88) = 7.941, p = 0.01, $\eta_{\text{p}}^{2}=0.265$) and stimulus type (F(1,22) = 29.6, p < 0.001, $\eta_{\text{p}}^{2}=0.574$) for reaction time was found. Reaction time was significantly shorter in the cycling condition than in the sitting condition (639.6 ± 107.9 ms vs. 663.1 ± 118.9 ms). For stimulus type, pairwise comparisons, indicated a shorter reaction time on the neutral stimuli (578.5 ± 85.8 ms) than on the colour congruent (621.4 ± 117.0 ms, p = 0.039), colour incongruent (757.4 ± 193.3 ms, p < 0.001), word congruent (613.6 ± 89.7 ms, p = 0.008) and word incongruent (685.6 ± 109.3 ms, p < 0.001) stimuli. Furthermore, reaction time was shorter on the colour congruent and word congruent stimuli than on the colour incongruent and word incongruent stimuli (p < 0.001). When comparing the interference scores for both conditions, no effect was observed. Mean ± SD values for reaction time and accuracy for both conditions and per stimuli type can be found in Table 2. No significant interaction effect for N200, P300, N450 and conflict SP latency and amplitude was observed. For N200 amplitude, a main effect of stimulus type was observed (F(2.438, 24.380) = 3.711, p = 0.032, $\eta_{\text{p}}^{2}=0.271$). However, further pairwise comparisons did not reveal significant differences between the different stimulus types. A main effect of stimulus type was also observed for P300 latency (F(4,44) = 4.458, p = 0.004, $\eta_{\text{p}}^{2}=0.288$). Further pairwise comparisons show the P300 latency to be longer for the neutral stimuli (394.7 ± 38.7 ms) than for the word congruent (358.8 ± 32.6 ms, p = 0.020) and word incongruent stimuli (358.4 ± 33.6 ms, p = 0.016). For the conflict SP, a main effect of stimulus type (F(4,48) = 2.593, p = 0.048, $\eta_{\text{p}}^{2}=0.178$). However, further pairwise comparisons did not reveal significant differences between the different stimulus types. Grand averaged stimulus-locked ERP waveforms in the frontal and the parietal region per stimulus type can be found in Fig 1.

### Rosvold continuous performance test

**Fig 2:**
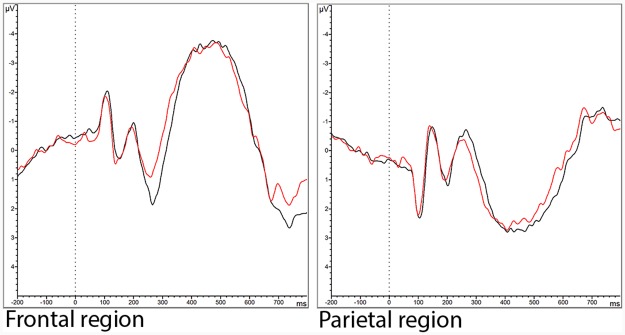
Grand averaged stimulus-locked ERP waveforms in the frontal (Fp1, Fp2, F4, F3, Fz, F7, F8, FC1, FC2) and the parietal (P3, P4, Pz, P7, P8, PO9, PO10) region in response to the Rosvold Continuous Performance test in the sitting (black line) and the cycling (red line) condition.

Accuracy did not significantly differ between the sitting and the cycling condition. Reaction time was shorter during cycling relative to sitting (377.9 ± 132.8 ms vs. 404.3 ± 174.6 ms; F(1,22) = 50.496, p < 0.001, $\eta_{\text{p}}^{2}=0.697$). Mean ± SD values for reaction time and accuracy for both conditions can be found in Table 2. No significant effect of condition on N200 or P300 latency or amplitude was observed. Grand averaged stimulus-locked ERP waveforms in the frontal and the parietal region per stimulus type can be found in Fig 2.

## Discussion

The aim of this study was to assess the participants’ typing and cognitive performance while cycling and sitting, using a protocol which included two familiarisation sessions, and to detect possible underlying neuronal processes. Therefore, event-related potentials N200 and P300 were investigated, with special focus on the fronto-central region for the N200 and the temporal-parietal region for the P300 [18, 36]. During the cycling condition, participants cycled at a power output of 55.3 ± 13.6 Watt and had a heart rate and RPE of 116.8 ± 16.0 bpm and 3.3 ± 1.1 respectively. This exercise intensity was higher than in the studies of Elmer et al. [12] and Straker et al. [10] (38 ± 14W; 5 and 30 W), but similar to the intensities used in the studies of Commissaris et al. [13], Koren et al. [11] and Pontifex et al. [18] (56 ± 21 and 85 ± 28 W; 40 and 80 W; 60% HRmax).

It is clear that reducing work-associated sitting behaviour could be an important means to reduce people’s overall sitting time and associated health risks, e.g. by the implementation of bike desks. However, there might be concerns about the influence of using bike desks on work productivity. Previous active workstation research used typing and cognitive tests to measure work performance. In the current study, typing performance did not significantly differ between the sitting and the cycling condition. This shows that, after two familiarisation sessions, people became accustomed to the combination of cycling at low intensity and performing a fine motor skill like typing. Earlier studies about the influence of cycling at low intensity on typing performance show contradictory findings. Straker et al. [10] and Koren et al. [11] reported a slightly worse typing performance when cycling at 5, 30, 40 and 80 Watt relative to rest. Results of the more recent studies of Elmer et al. [12] and Commissaris et al. [13], show no detrimental effect of cycling at low intensity (38 ± 14 W; 56 ± 21 and 85 ± 28 W) on typing performance. This is in line with the findings of the current study. The present study also shows that low intensity cycling does not affect short-term memory. Previous research concerning the influence of low intensity exercise, more specifically treadmill walking, on short-term memory is inconclusive. Larson et al. [19] reported a negative effect of using a treadmill desk on the RAVLT, while Ohlinger et al. [15] did not find any effect on the Auditory Consonant Trigram Test.

In the current study improved performances on the Stroop test and on the RCPT were found. This was demonstrated by a shorter reaction time during cycling at low intensity, relative to sitting. The improvement in reaction time on the RCPT indicates an increased sustained attention. On the Stroop test, we found an overall improvement in reaction time. However, to confirm an improvement in selective attention and response inhibition due to the cycling, a difference in interference scores between the cycling and the sitting condition would be expected. Based on the current results, we can conclude that improvements in task performance were not stimulus-specific and thus independent of the difficulty level. Selective attention, response inhibition and sustained attention during low intensity exercise have previously been studied during both treadmill walking and cycling [9, 13, 17]. These previous studies showed no significant effect of low intensity exercise on selective attention and response inhibition, measured with the Stroop and the Flanker test [9, 13, 17]. Furthermore, they did not find any effect on sustained attention and response inhibition, measured with a Go/No-go task [13, 17]. The inclusion of several familiarisation sessions in the current protocol is a possible reason for these different findings on measures of cognitive performance, as familiarisation trials in the previous studies were limited, with the longest familiarisation period being 15 minutes [9, 12, 13, 17, 19]. This might have led to measuring the effect of being new to an unknown dual task, during which cycling or walking still requires a large part of the cognitive capacity and thus could interfere with other cognitive tasks, instead of measuring the actual effect of walking or cycling on cognitive performance [38].

The evaluation of ERP’s provides additional insight into underlying mechanisms involved in cognitive function beyond that of behavioural measures. Only few studies looked into ERP’s during cognitive tests performed while cycling. Pontifex et al. [18] observed a reduced response accuracy for incongruent trials but no effect on congruent trials on the Flanker test when cycling at 60% HRmax relative to rest. Cycling at 60% HRmax resulted in a globally decreased N200 amplitude, an increased P300 amplitude at frontal and lateral sites, and longer N200 and P300 latencies at central-parietal and parietal sites, relative to rest. The findings of the latter study [18] suggest that the need to allocate attentional resources toward the bodily movements associated with exercise may relate to inefficiency of neural resource allocation, resulting in decreased interference control. In the current study, exercise seemed to have a facilitating effect on cognitive performance. However, this improvement in performance on the cognitive tests was not associated with changes in brain activity. Reaction time can be fractioned into the two components premotor time and motor time [39]. The premotor time reflects the period in which information processing takes place whereas the motor time reflects the electromechanical transduction within muscular fibres. It might be that cycling influenced motor time, but not premotor time. This would mean that the improvement in reaction time can be explained by peripheral mechanisms rather than by an alteration of cognitive processes, which would also explain why overall reaction time on the Stroop test improved similarly as the reaction time on the RCPT, but no interference effects were seen.

This study is the first one to use a longer familiarisation exposure to the cycling task and the cognitive tasks. We demonstrated that people are able to work on a bike desk with equal typing performance and short-term memory and improved response speed across tasks requiring variable amounts of attention and inhibition. Implementation of these workstations could not only contribute to interrupting people’s sitting behaviour in for example an office setting, and help reducing the adverse health effects of sedentary behaviour, but could also improve people’s cognitive performance and thus possibly work performance. Therefore, this study provides positive evidence for the implementation of bike desks in offices. However, in this study, a time span of approximately 30 minutes of cycling while performing tasks was assessed. Consequently, no conclusions about the use of the bike desk for a longer period of time can be made. Additionally, longitudinal studies looking at the effect of the regular use of these desks on health parameters, cognitive function and work performance are needed to establish the long-term effects of using bike desks to interrupt sitting behaviour. Furthermore, research about people’s compliance to using a bike desk is needed.

## Conclusion

This study shows that cycling at 30% Wmax on a bike desk does not influence typing performance and short-term memory. Moreover, it has a positive effect on response speed across tasks requiring variable amounts of attention and inhibition. These findings suggest that implementing bike desks in office settings could not only contribute to reducing health risks associated with excessive sitting, but could also contribute to an improved cognitive performance, therefore work performance.
